# Association between Psychopathological Dimensions and Sexual Functioning/Sexual Arousal in Young Adults

**DOI:** 10.3390/ijerph18073584

**Published:** 2021-03-30

**Authors:** Franklin Soler, Reina Granados, Ana I. Arcos-Romero, Cristóbal Calvillo, Ana Álvarez-Muelas, María del Mar Sánchez-Fuentes, Nieves Moyano, Juan Carlos Sierra

**Affiliations:** 1 School of Medicine and Health Sciences, Universidad del Rosario, Bogotá 111221, Colombia; 2 Facultad de Ciencias de la Salud, Universidad de Granada, 18016 Granada, Spain; reina@ugr.es; 3 Department of Psychology, Universidad Loyola, 41704 Sevilla, Spain; aiarcos@uloyola.es; 4 Centro de Investigación Mente, Cerebro y Comportamiento (CIMCYC), Universidad de Granada, 18011 Granada, Spain; ccalvillo@correo.ugr.es (C.C.); alvarezm@ugr.es (A.Á.-M.); jcsierra@ugr.es (J.C.S.); 5 Facultad de Ciencias Sociales y Humanas, Universidad de Zaragoza, 44003 Teruel, Spain; marsanchez@unizar.es; 6 Facultad de Humanidades y Ciencias de la Educación, Universidad de Jaén, 23071 Jaén, Spain; mnmoyano@ujaen.es

**Keywords:** sexual functioning, sexual arousal, psychopathology, young adults

## Abstract

Psychological-psychiatric factors have a different effect on sexual functioning in men and women. This research aimed to examine the association between psychopathological dimensions and dimensions of sexual functioning in Spanish young adults in two studies. Study 1 examined sexual functioning and psychopathological dimensions in 700 women and 516 men. Study 2 conducted an experimental laboratory task to evaluate subjective sexual arousal and genital sensations when watching visual sexual stimuli in a subsample of participants from Study 1 (143 women and 123 men). As a result, the first study showed that depression and anxiety-related symptoms had a negative effect, both in men and women, and having a partner had a positive influence on the dimensions of sexual functioning. The second study showed that anxiety symptoms were positively associated with subjective sexual arousal in both men and women, and anxiety was associated with the assessment of genital sensations in men. The differences between the results of anxiety may be explained because sexual arousal was evaluated in general terms in Study 1, whereas it was evaluated as a state in Study 2. These findings confirm that the presence of psychopathological symptoms contributes to sexual functioning, as well as the necessity of strengthening mental illness prevention programs that include sexual health components.

## 1. Introduction

Sexual dysfunction is a diagnostic category used to refer to alterations in the functioning of the sexual response, which includes difficulties related to sexual desire, arousal, orgasm, pelvic or genital pain, erection, and ejaculation [1]. Regarding the prevalence of sexual dysfunctions, this is higher in women and varies between 20% and 70% [2,3,4], while in men, percentages oscillate between 15% and 35% [5,6].

The risk factors for sexual dysfunction include biological, psychological-psychiatric, and sociocultural factors [7]. The influence of these factors differs depending on gender [8,9]. The prevalence of sexual dysfunctions is higher among the population diagnosed with mental illness [10], and they become more intense depending on the severity of the disorder [11,12,13]. Alterations in sexual functioning have been identified in patients with schizophrenia [14,15], depression [13], anxiety [16,17], obsessive-compulsive disorder [17,18], post-traumatic stress [19,20], bipolar disorder [21], eating disorders [22,23], and personality disorders [24,25]. Consumption of psychotropic drugs, especially some antipsychotics and antidepressants, induces or worsens sexual dysfunctions [26,27].

The prevalence of sexual dysfunctions has also been observed in general (i.e., nonclinical) population. In Spain, the most prevalent dysfunctions in men and women are those related to desire, arousal, and orgasm [28]. These sexual dysfunctions have been found to increase with age [28]. Studies on alterations in sexual functioning in young adults are scarce, even though they also experience sexual dysfunctions. Following Arnett’s proposal [29], and in accordance with the subjective perception of adult status, this study considered the two youngest ranges of adulthood, that is, 18 to 25 years and 26 to 35 years old.

There is a lack of research on the implication of psychopathological symptoms on sexual functioning among non-clinical samples. The main objective of the present work was to analyze sexual functioning by using self-reported measures as well as conducting an experimental study in a laboratory. To do so, two studies were carried out. Their specific objectives were the following:

1. To examine the association between psychopathological dimensions and dimensions of sexual functioning (i.e., desire, arousal, erection, orgasm, and sexual satisfaction).
2. To examine the association of psychopathological dimensions with a specific dimension of sexual response within a laboratory task (i.e., subjective sexual arousal).

## 2. Study 1

### 2.1. Materials and Methods

#### 2.1.1. Participants

Using a non-probabilistic sampling method, a sample of 1216 Spanish participants (42% men and 58% women) with ages between 18 and 35 years (M = 21.40; SD = 3.42) was recruited. Participants answered a Background Questionnaire and Sexual History, which assessed information about gender, age, nationality, having a partner, age of the first sexual relationship, number of sexual partners, and sexual orientation. The inclusion criteria were: (a) Spanish nationality and (b) being between 18 and 35 years old. Table 1 shows the sociodemographic characteristics of the sample by gender. The strength of the statistical analysis had a small-to-medium effect size.

#### 2.1.2. Instruments

Background Questionnaire and Sexual History. Described in Participants’ section.

Spanish version of the Symptom Assessment-45 Questionnaire (SA-45) [30] by Sandín et al. [31]. Through 45 items, this questionnaire assesses different symptoms grouped into nine psychopathological categories: Hostility, Somatization, Depression, Obsession-compulsion, Anxiety, Interpersonal Sensitivity, Phobic Anxiety, Paranoid Ideation, and Psychoticism. Each item is answered on a Likert-scale with five alternatives (from 0 = not at all to 4 = a lot or extremely) that measure the intensity with which the symptoms are experienced. Higher scores indicate worse mental health. This instrument has shown good indices of internal consistency, and discriminant and convergent validity [31]. It is noteworthy that the scores obtained in the SA-45 Questionnaire do not reveal the presence of clinically significant symptoms. In the present study, the ordinal alpha was between 0.70 (Psychoticism) and 0.88 (Anxiety).

Spanish version of the Massachusetts General Hospital Sexual Functioning Questionnaire (MGH-SFQ) [32] by Sierra et al. [28]. It is composed of five items that evaluate sexual functioning at the present time through five dimensions: Desire, Arousal, Orgasm, Erection (in men), and Satisfaction. The items are answered on a Likert-type response scale with five alternatives from 0 = totally diminished to 4 = normal. Normal refers to the period of life when the person has been most satisfied with their sexual functioning. Higher scores indicate better sexual functioning, whereas low scores indicate the possible presence of problems in these dimensions. Thus, no specific sexual dysfunctions were assessed. The Spanish version has shown good psychometric properties [28]. In the present study, the ordinal alpha was 0.84 for men and 0.90 for women.

#### 2.1.3. Procedure

Since this research aims to understand the relationship between the dependent and independent variables that occurred, this study is correlational. Participation in this study was voluntary without compensation. All the self-reported measures were answered online through the LimeSurvey platform. The access URL was disseminated through the social networks of Laboratorio de Sexualidad Humana LabSex UGR. Participants were recruited between March 2017 and December 2019. By controlling the IP address, repeated responses were avoided. To prevent automatic responses, participants had to confirm access to the survey by answering a security question consisting of a simple random arithmetic operation. When accessing the survey, participants were directed to the informed consent form, the participation form, and the guarantee of anonymity and confidentiality of the data. Once they agreed to take part in the study, participants were then directed to the self-reported measures. The study was approved by the Ethics Committee of the University of Granada.

#### 2.1.4. Data Analysis

A multivariate exploratory analysis was carried out. It was observed that some of the variables did not meet the univariate normality criterion, and therefore non-parametric statistics were used for these cases. The Mann–Whitney *U* test or chi-squared test were used to evaluate the differences between genders depending on the nature of the data. The associations between the dimensions of sexual functioning with the continuous and categorical sociodemographic variables were calculated with the Pearson correlation coefficient and with Mann–Whitney U test, respectively. R software Version 3.6.3 [33] and the lavaan package Version 0.6–4 [34] were used to perform linear regressions using structural equation models (SEM) [35]. Compliance with the assumptions of linearity, heteroscedasticity, residual independence (the Durbin–Watson index in all models was between 1.5 and 2.5, and the condition indices were less than 5), and multicollinearity (VIF < 2) was previously verified [36]. To identify the best explanatory variables, the stepwise method was used for the inclusion of variables in each model. Due to the properties of the variables, the estimation method used for the regression analysis with SEM was the Weighted Least Squares (WLS) [37,38]. The effect size was used to assess the strength of each analysis. The Mann-Whitney *U* test and chi-squared test were measured with Cohen’s d (small 0.2; medium 0.5; large 0.8; very large 1.3), and the regression models with the coefficient of determination R^2^ (small 0.04; medium 0.25; large 0.64) [39].

The listwise deletion technique was used to manage the missing data for sociodemographic variables. This accounted for less than 3% of the sociodemographic information. In addition, to control the missing data for the measures of the psychopathological and sexual functioning variable dimensions, we counted values within cases for each examined dimension and calculated the percentages of missing data. Only those participants who had answered at least 75% of the items were included, proceeding to replace the missing values using the “median of nearby points” method with the total amplitude of the points [40]. In this method, the span of nearby points is the number of valid values above and below the missing value used to compute the median [41].

### 2.2. Results

First, differences between men and women were examined in the observed variables: psychopathological dimensions and sexual functioning (see Table 2).

Men showed significantly higher scores in sexual excitation (*z* = −5.38, *p* < 0.001) and orgasm (*z* = −8.38, *p* < 0.001). Women scored significantly higher in interpersonal sensitivity (*z* = 2.68, *p* < 0.01), somatization (*z* = 30.49, *p* < 0.001), anxiety (*z* = 4.56, *p* < 0.001), and phobic anxiety (*z* = 2.59, *p* < 0.05). Overall, the effect size of the differences between men and women was small.

No significant correlations were found between the sociodemographic variables age, age of first sexual relationship, and number of sexual partners in men. In women, age was significantly correlated with sexual desire (*r* = −0.09; *p* < 0.05). Table 3 shows the associations between the dimensions of sexual functioning and sexual orientation and having a partner in men and women. The variables that had a significant association with the sexual functioning dimensions were included as explanatory variables in the regression models.

Considering that half of the analyzed variables showed statistically significant differences between men and women, as well as the inclusion of the erection item to measure a part of male sexual functioning, we decided to perform a hierarchical linear regression analysis of sexual functioning in men and women, separately. Results showed that all the models were significant and presented adequate goodness of fit. The models that explain the statistically significant amount of variance in the different variables are presented in Table 4.

Anxiety was negatively associated with sexual desire and arousal in men, while having a partner was positively associated them. Desire was also negatively associated with somatization. Orgasm was negatively associated with psychoticism and phobic anxiety. Sexual satisfaction was negatively associated with depression and phobic anxiety, whereas it was associated with having a partner. Finally, erection was negatively associated with depression and paranoid ideation and positively associated with hostility.

In women, depression—negatively-, and having a partner—positively-, were the most consistent variable of all dimensions (desire, arousal, orgasm, and sexual satisfaction). Desire was negatively associated with age, and sexual satisfaction was also negatively associated with psychoticism.

The explanatory models of sexual functioning, in both men and women, had a small-to-medium effect. The variables analyzed explained 11.9% of sexual satisfaction in men and 16.5% in women, this dimension being the one that had the best effect.

**Figure 1:**
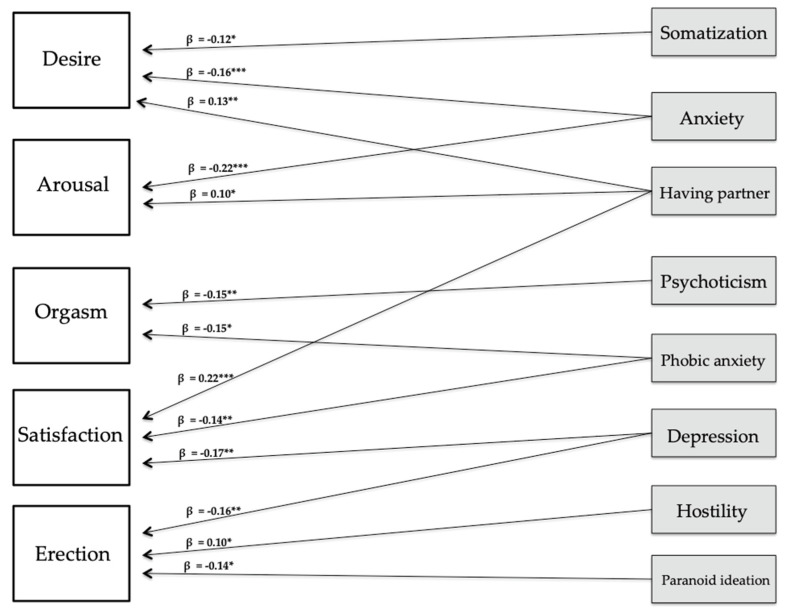
Graphical representation of the SEM in men. *** *p* < 0.001; ** *p* < 0.01; * *p* < 0.05.

**Figure 2:**
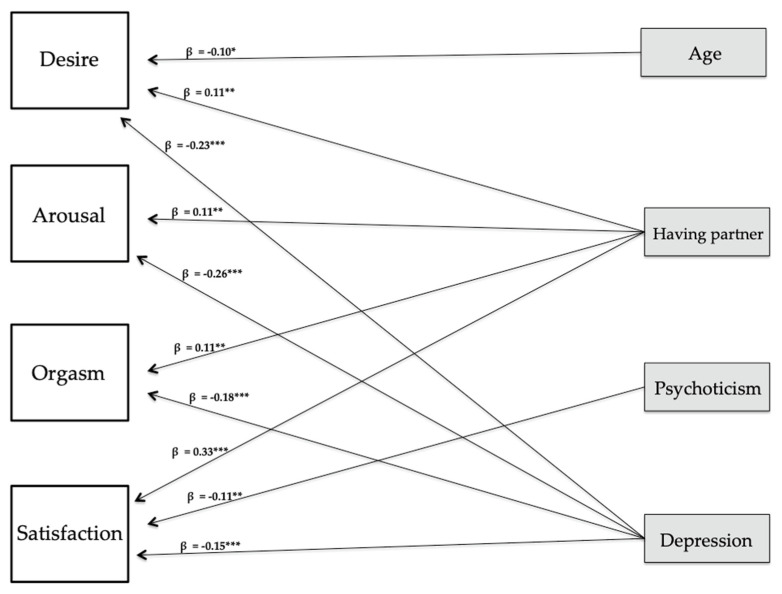
Graphical representation of the SEM in women. *** *p* < 0.001; ** *p* < 0.01; * *p* < 0.05.

The results of the graphical representation of the SEM are presented in Figure 1 for men and in Figure 2 for women.

#### Discussion Study 1

The objective of this study was to examine to what extent psychopathological symptoms explained sexual functioning in men and women. As expected, and since it is a general population sample, the high scores obtained in the psychopathological dimensions and the low scores obtained in sexual functioning did not show the possible presence of significant clinical symptoms, or the presence of severe sexual dysfunctions. Men reported better sexual functioning than women, according to what has been reported in previous literature [28,42,43,44]. In general, despite some differential nuances across gender, it can be noted that depression- and anxiety-related symptoms had a negative association, and having a partner had a positive association on sexual functioning. They are the most consistent explanatory variables.

## 3. Study 2

The objective of this study was to specifically analyze if psychopathological symptoms explain the subjective sexual arousal experienced by young adults when watching visual sexual stimuli within a laboratory setting.

### 3.1. Materials and Methods

#### 3.1.1. Participants

The following criteria were considered for participants’ inclusion in the study: (a) heterosexual orientation, (b) not having medical problems, (c) not indicating consumption of medication (e.g., antidepressants, antihypertensive), drugs, or alcohol abuse, and (d) not having suffered a history of sexual abuse. After considering the inclusion criteria, a subsample of participants from Study 1 was invited to participate in an experimental task conducted in a laboratory setting. This sample consisted of 266 Spanish heterosexual young adults (46% men and 54% women), with ages between 18 and 32 years old (M = 20.90; SD = 2.53). Table 5 presents the sociodemographic characteristics of the sample by gender.

#### 3.1.2. Procedure

This study used an experimental design since its purpose was to explain the association between psychopathological symptoms and the subjective sexual arousal experienced. In order to carry out this study, participants from Study 1 were assigned an alphanumeric code that was associated with their e-mail address and telephone number. Then, through a telephone call, participants who met the inclusion criteria for this study were invited to participate in an experimental task conducted in a laboratory setting. Once they indicated an interest in participating in the study, a date was set for them to go to the Laboratorio de Sexualidad Humana LabSex UGR of the University of Granada. Participants were informed, via e-mail, of the goal of the study, the procedures to be carried out, what their participation would entail, their volunteer nature, anonymity, and confidentiality of their data. Then, when the participant was in the laboratory, the informed consent was signed. Participants had to remain alone in an isolated room whose temperature, noise level, and lighting remained constant. The experimental task consisted in watching two films: (a) a three-minute neutral content film (nature documentary); (b) a three-minute erotic content film (heterosexual couple having a sexual relationship including oral sex—cunnilingus and fellatio—and vaginal intercourse). Taking into account the design of the experimental task conducted in a laboratory setting, the visualization of the neutral content film serves as an adaptive phase before the assessment of sexual arousal; while the erotic film used had previously been shown to induce sexual arousal in men and women in a pilot study [45]. The films were projected on a 24” screen. At the end of the sexual film, the participant answered the Ratings of Sexual Arousal and the Ratings of Genital Sensations scales in paper and pencil format. Through the alphanumeric code, the answer of Symptom Assessment-45 Questionnaire was associated with the evaluation of sexual excitation in the experimental task. The study was approved by the Ethics Committee of the University of Granada.

#### 3.1.3. Instruments

Spanish version of the Symptom Assessment-45 Questionnaire (SA-45) [30] by Sandín et al. [31]. Described in Study 1. Participants answered this questionnaire in Study 1.

Spanish version of the Ratings of Sexual Arousal (RSA) [46], included in the Multiple Indicators of Subjective Sexual Arousal [47]. It evaluates subjective sexual arousal through five items on a Likert-scale (from 1 = no arousal to 7 = extremely aroused). Higher scores indicate a higher degree of subjective sexual arousal. Sierra et al. [46] reported good reliability and validity indices. In the present study, the ordinal alpha was 0.88.

Spanish version of the Ratings of Genital Sensations (RGS) [46], included in the Multiple Indicators of Subjective Sexual Arousal [47]. It is composed of a list of 11 levels of genital sensations that range from no genital sensation (0) to multiple orgasms (11). Higher scores indicate a greater degree of subjective sexual arousal through genital sensations. Sierra et al. [46] have previously provided its validity evidence.

Visual stimuli materials. Described in the Procedure section.

### 3.2. Results

First, the examined variables were compared by gender. Women scored significantly higher in interpersonal sensitivity (*z* = 2.70, *p* < 0.01), anxiety (*z* = 3.59, *p* < 0.01) and assessment of their genital sensations (*z* = 2.36, *p* < 0.05). Overall, the effect size of the differences between men and women was small. Besides, the association between sociodemographic characteristics and subjective sexual arousal were examined. There was no significant association and, for this reason, the sociodemographic characteristics were not included in the regression analyses (see Table 6).

The regression models were estimated following the same method of the previous study to determine the explanatory variables of RSA and RGS, with had a small effect size. Results showed that in both men and women, RSA was explained by anxiety. Only the female model was significant. For the explanatory models of RGS, the variables with explanatory capacity were anxiety, phobic anxiety, and compulsion obsession in men. No significant explanatory model was found in women (see Table 7).

#### Discussion Study 2

This study aimed to determine whether psychopathological symptoms explain subjective sexual arousal in a sample of young adults. Our findings indicate that anxiety symptomatology explains subjective sexual arousal in women, similarly to previous studies [48]. Although the explanatory model of subjective sexual arousal and the explanatory model of genital sensations in men were not significant, a significant positive association of anxiety and a negative association of phobic anxiety and obsessive compulsion were observed. A possible reason to be taken into consideration for the lack of significance of explanatory models in men may be the fact that sexual arousal was evaluated as a state in the exposure to visual sexual stimuli. However, there is no psychopathological association on how women value their genital sensations.

## 4. Discussion

The general objective of the present research was to analyze the association of psychopathological symptoms with sexual functioning and its specific dimensions in a general population of young adults. To do so, two interdependent studies were carried out. The first aimed to examine the association of psychopathological symptoms with different sexual functioning dimensions (i.e., desire, excitation, orgasm, and sexual satisfaction). The second aimed to specifically examine the association of psychopathological symptoms with the subjective sexual arousal experienced within a laboratory context.

Differences across gender were observed. Women reported a greater number of psychopathological symptoms, and worse sexual functioning than men. However, we cannot determine, for any of the dimensions, whether these are clinically significant symptoms. Regarding sexual functioning dimensions, women reported lower excitation and orgasmic capacity. These findings are in line with previous studies that indicate that women report overall worse sexual functioning, with greater difficulties specifically in the responses to orgasm and sexual desire/excitation [28,42,44,49], and less sexual satisfaction than men [50,51]. Therefore, women show more sexual problems [28] and sexual dysfunctions [52]. These results are congruent with previous findings from the study by O’Sullivan et al. [43], conducted in a young non-clinical population.

The regression models have shown that psychopathological symptoms explained a small significant percentage of the variance of the sexual functioning dimensions, in accordance with previous studies [53,54]. These results seem logical because: first, participants were young adults recruited from the general population; and second, in addition to mental illness symptoms, the psychological variables that also affect sexual functioning include concerns, distress, sexual beliefs, automatic negative thoughts, cognitive schemas, emotions, and attitudes toward sexuality [55,56]. And those variables were not analyzed in this study.

In the explanatory models, depressive symptoms proved to be the most relevant variables associated with all sexual functioning dimensions in women, and with satisfaction and erection in men. This result is congruent with other studies that have found that depression negatively affects sexual functioning in men and women [4,16,57,58,59,60,61]. In addition, having a partner was associated with all sexual functioning dimensions in both genders, except in orgasm and erection in men. These findings are consistent with the previous study by Malakouti et al. [62], which indicated that having a partner is positively associated with sexual functioning in men and women.

In men, anxiety-related symptoms negatively and significantly affected desire, sexual arousal, orgasm, and satisfaction [63,64,65,66,67,68,69]. This could be due to a feedback loop in which, due to anticipatory fear, arousal responses are inhibited, or sexual performance worsens, accelerating possible orgasm and ejaculation problems [66,70]. In addition to anxiety, somatization were significant explanatory variables of male sexual desire, according to previous findings [71].

In women, age was found to have a negative association with the desire dimension. Previous evidence has found that age has a negative impact on women’s sexuality [72].

Sexual satisfaction is related to sexual functioning dimensions and individual variables such as physical and mental health, and relational variables such as satisfaction are related to the couple’s relationship or the type of relationship [68,73,74]. This may explain why it was the most affected dimension in both men and women. In men, depression and anxiety symptoms were negative explanatory variables of sexual satisfaction, which had already been reported [74,75,76]. In the case of women, sexual satisfaction was explained, as had already been demonstrated, by the presence of depressive symptoms [64,74,77] and psychoticism [78]. On the other hand, having a partner was found to explain the greatest sexual satisfaction in men and women. This could be due to factors such as duration of the couple relationship, or satisfaction with the couple relationship, the latter of which was shown to have a greater impact on explaining satisfaction in heterosexual and same-sex couples [79,80,81]. However, more research is needed in this regard. On the other hand, our results found that satisfaction was negatively affected by psychoticism in women. Sexual satisfaction was found to be greater with a better mental health balance [68,69].

Erection was the dimension that presented the highest scores, which confirms that alterations in this dimension are not common in the young population [6], which is negatively affected by depression and paranoid ideation and, positively affected by hostility. These results agree with the evidence of an increase in erectile dysfunction problems in the presence of depressive symptoms [82]. It should be noted that the paranoid ideation and hostility variables have not been consistently demonstrated or studied. However, hostile personality traits have been reported to not affect erection [83].

Regarding subjective sexual arousal to erotic stimuli in an experimental laboratory task, women reported higher levels of sexual arousal through genital sensations in contrast with Study 1, where women reported lower levels of sexual excitation than men. This inconsistency between results in Study 1 and Study 2 may be explained because sexual arousal was evaluated in general terms in Study 1, whereas sexual arousal was evaluated as a state in the exposure to visual sexual stimuli in Study 2. In the first study, depression was the explanatory variable for women. These findings are in line with studies reporting that the ability to achieve sexual arousal is negatively affected by depressive symptomatology [13,61]. In the case of men, it was affected by anxiety. This finding is similar to the Corretti and Baldi study [63]. Therefore, for men and women, RSA was significantly and positively associated with anxiety, while RGS was significantly positively associated with anxiety and negatively with obsessive-compulsive symptoms and phobic anxiety [84] in men. Although the RSA was only significant in women, and RGS was not significant in men or women, a significant effect of anxiety was evidenced, reflecting a discrepancy that has not yet been resolved: anxiety may favor the sexual arousal of healthy men [85] and women [48]. Specifically, moderate levels of anxiety could facilitate sexual arousal in healthy women [85,86], but not in women with a sexual arousal dysfunction [87,88]. These discrepancies may be related, on the one hand, to the fact that sexual arousal is a category that involves physiological and subjective components, whose measurements may be discordant [89], and on the other hand, to the type of anxiety (e.g., trait or state), the instruments used to account for it, and the experimental strategies applied to evaluate it [66,87].

As limitations, since an incidental non-probabilistic sampling method was used, our findings cannot be generalized to the Spanish population of young adults. For future research, the measurement of other dimensions such as relational aspects or sexual satisfaction from a broader and multidimensional perspective is recommended [90,91]. It is also advisable to make a more specific evaluation of the symptoms of anxiety, as this is a relevant variable for sexual functioning. Furthermore, the possible effects of other risk factors (e.g., endocrine dysfunctions [92], erectile dysfunction [93], smoking habits [94]) that could be associated with sexual functioning aspects have not been examined. Previous research stated that healthy lifestyle variables are related to both male and female sexuality [95]. Finally, this study did not assess the use of pornography; therefore, this variable was not taken into account. However, some studies have shown that, generally speaking, men are more interested in pornography than women [51,96,97]. This information could be relevant when using erotic content films to assess subjective sexual arousal.

## 5. Conclusions

According to previous studies [58,64,77,98,99], our findings indicate that the symptoms of depression and anxiety, have the greatest effect, and are the best explanatory variables of general sexual functioning and its different dimensions. These results confirm that the presence of psychopathological symptoms contributes to sexual functioning [12,100]. Therefore, these findings could be used to support future research about psychopathological symptoms and sexual functioning in the clinical field. The symptoms of depression and anxiety could be taken into account in sexual health programs, especially the evaluation and treatment of sexual functioning. In this way, it is necessary to strengthen mental illness prevention programs that include sexual health components and strategies to evaluate sexual functioning in young people.

## Figures and Tables

**Table 1:** Sociodemographic characteristics of the sample from Study 1.

Variable	Men (n = 516)	Women (n = 700)	U/χ^2^	Cohen′s d
	M (SD)/n (%)	M (SD)/n (%)		
Age	22.14 (3.76)	20.93 (3.05)	143,573 *	0.35
Having a partner				
Yes	227 (44%)	409 (58%)	26.92 *	0.30
No	271 (53%)	263 (38%)		
Age of first sexual relationship	16.42 (2.97)	16.13 (1.74)	137,149 *	0.42
Number of sexual partners	8.3 (15.19)	5.9 (8.52)	147,527.50 *	0.32
Sexual orientation				
Heterosexual	388 (75%)	603 (86%)	23.59 *	0.14
Non-heterosexual	123 (24%)	92 (13%)		

U: Mann-Whitney U test; χ^2^: chi-squared. * *p* < 0.01.

**Table 2:** Differences between men and women in psychopathological dimensions and sexual functioning.

Variables	Range	Men (n = 516)	Women (n = 700)	U	Cohen′s d
		M (SD)	M (SD)		
Psychopathological dimensions					
Depression	0–20	5.26 (3.92)	5.36 (4.17)	180,479.50	
Hostility		2.21 (2.63)	2.26 (2.86)	178,151.50	
Interpersonal sensitivity		4.23 (3.57)	4.78 (3.78)	196,722 **	0.15
Somatization		2.53 (2.70)	3.09 (2.92)	201,473.50 ***	0.19
Anxiety		3.65 (3.09)	4.51 (3.42)	208,000.50 ***	0.26
Psychoticism		1.47 (1.93)	1.29 (1.75)	173,687.50	
Obsession compulsion		4.97 (3.62)	5.17 (3.64)	186,821.50	
Phobic anxiety		1.22 (1.94)	1.60 (2.41)	195,165.50 *	0.13
Paranoid ideation		4.55 (3.37)	4.58 (3.28)	181,975	
Sexual functioning					
Desire	0–4	3.38 (1.03)	3.30 (1.08)	173,448.50	
Excitation		3.63 (0.79)	3.34 (1.04)	154,256 ***	0.25
Orgasm		3.72 (0.72)	3.22 (1.20)	140,049 ***	0.39
Satisfaction		3.02 (1.25)	3.01 (1.31)	183,314.50	
Erection		3.74 (0.62)			

U: Mann-Whitney U test. *** *p* < 0.001; ** *p* < 0.01; * *p* < 0.05.

**Table 3:** Mann-Whitney *U* test for associations between sociodemographic characteristics and dimensions of sexual functioning.

	Men		Women	
	Sexual Orientation	Having a Partner	Sexual Orientation	Having a Partner
Desire	26,989.50 **	33,506.50 *	30,389.00	58,918.50 *
Arousal	26,048.00 *	32,815.00	30,010.00	59,884.00 **
Orgasm	24,409.50	31,185.00	26,914.50	59,403.50 **
Satisfaction	25,854.00	37,498.00 ***	27,696.50	71,892.00 ***
Erection	23,569.00	31,939.00		

*** *p* < 0.001; ** *p* < 0.01; * *p* < 0.05.

**Table 4:** Hierarchical linear regression analysis of the dimensions of sexual functioning and of the explanatory role of psychopathological dimensions and sociodemographic characteristics in men and women.

Variable		β	Std. Err	*z*-Value	*R* ^2^	χ^2^
Men						
Desire						
	Somatization	−0.12	0.019	−2.48 *	0.074	34.14 ***
	Anxiety	−0.16	0.015	−3.63 ***		
	Having a partner	0.13	0.088	3.07 **		
Arousal						
	Anxiety	−0.22	0.013	−4.44 ***	0.058	21.51 ***
	Having a partner	0.10	0.072	2.36 *		
Orgasm						
	Psychoticism	−0.15	0.020	−2.85 **	0.059	13.03 **
	Phobic anxiety	−0.15	0.024	−2.41 *		
Satisfaction						
	Depression	−0.17	0.016	−3.28 **	0.119	56.46 ***
	Phobic anxiety	−0.14	0.032	−2.82 **		
	Having a partner	0.22	0.11	5.08 ***		
Erection						
	Depression	−0.16	0.010	−2.82 **	0.056	15.20 **
	Hostility	0.10	0.011	2.03 *		
	Paranoid ideation	−0.14	0.012	−2.18 *		
Women						
Desire						
	Depression	−0.23	0.011	−5.74 ***	0.073	40.42 ***
	Having a partner	0.11	0.086	2.77 **		
	Age	−0.10	0.017	−2.06 *		
Arousal						
	Depression	−0.26	0.010	−6.42 ***	0.086	41.40 ***
	Having a partner	0.11	0.082	2.99 **		
Orgasm						
	Depression	−0.18	0.012	−4.38 ***	0.048	23.47 ***
	Having a partner	0.11	0.096	2.80 **		
Satisfaction						
	Depression	−0.15	0.013	−3.80 ***	0.165	93.87 ***
	Psychoticism	−0.11	0.031	−2.65 **		
	Having a partner	0.33	0.10	8.57 ***		

R^2^: adjusted R-squared value; χ^2^: chi-square. *** *p* < 0.001, ** *p* < 0.01, * *p* < 0.05.

**Table 5:** Sociodemographic characteristics of the sample from Study 2.

Variable	Men (n = 123)	Women (n = 143)	U/χ^2^	Cohen′s d
	M (SD)/n (%)	M (SD)/n (%)		
Age	21.42 (2.67)	20.51 (2.28)	6998 *	0.36
Having a partner				
Yes	50 (41%)	94 (66%)	20.85 *	0.58
No	71 (58%)	47 (33%)		
Age of first sexual relationship	16.68 (1.69)	16.16 (1.44)	6338 *	0.50
Number of sexual partners	5.28 (6.63)	5.15 (6.52)	7937	

U: Mann-Whitney U test. * *p* < 0.01.

**Table 6:** Differences between men and women in the psychopathological dimensions, subjective sexual arousal, and assessment of genital sensations.

Variable	Range	Men (n = 123)	Women (n = 143)	U	Cohen′s d
		M (SD)	M (SD)		
Psychopathological dimensions					
Depression	0–20	4.68 (3.50)	4.50 (3.29)	8532.50	
Hostility		2.05 (2.54)	2.06 (2.46)	8906	
Interpersonal sensitivity		3.27 (3.00)	4.30 (3.28)	10,472 **	0.33
Somatization		2.28 (2.88)	2.75 (3.17)	9648.50	
Anxiety		2.85 (2.64)	3.93 (2.72)	11,024 **	0.44
Psychoticism		1.02 (1.40)	1.03 (1.58)	8550	
Obsessive compulsion		4.54 (3.26)	4.30 (3.14)	8421	
Phobic anxiety		0.70 (1.29)	1.03 (1.69)	9542	
Paranoid ideation		4.37 (3.43)	4.22 (2.88)	8845	
Global score SA-45	0–180	25.76 (16.10)	28.12 (17.62)	9311.50	
Sexual arousal					
Subjective sexual arousal	1–35	18.30 (6.59)	18.92 (5.47)	9353.50	
Assessment of genital sensations	1–11	3.20 (1.46)	3.55 (1.40)	10,222 *	0.28

U: Mann-Whitney U test. ** *p* < 0.01; * *p* < 0.05.

**Table 7:** Hierarchical linear regression analysis of subjective sexual arousal, and of the association of psychopathological dimensions.

Variables	Β	Std. Err	*z*-Value	R^2^	χ^2^
Subjective sexual arousal					
Men					
Anxiety	0.22	0.273	1.97 *	0.029	4.26
Women					
Anxiety	0.16	0.151	2.11 *	0.025	4.31 *
Assessment of genital sensations					
Men					
Anxiety	0.25	0.069	1.98 *	0.043	7.05
Phobic anxiety	−0.14	0.075	−2.10 *		
Obsessive compulsion	−0.22	0.045	−2.15 *		

R^2^: adjusted R-squared value; χ^2^: chi-square. * *p* < 0.01.

## Data Availability

The data presented in this study are available on request from the corresponding author. The data are not publicly available due to privacy.
